# Endomycorrhizal association of *Funneliformis mosseae* with transformed roots of *Linum usitatissimum*: germination, colonization, and sporulation studies

**DOI:** 10.1080/21501203.2015.1024777

**Published:** 2015-03-20

**Authors:** Kim Maria Rodrigues, Bernard Felinov Rodrigues

**Affiliations:** Department of Botany, Goa University, Goa, India

**Keywords:** *Funneliformis mosseae*, monoxenic culture, transformed linum roots, MSR, *in vivo*

## Abstract

Spores of arbuscular mycorrhizal (AM) fungus *Funneliformis mosseae* (Nicolson & Gerdemann) Walker & Schuessler were cultured in association with transformed roots of *Linum usitatissimum* L. (Linaceae) for the first time on modified Strullu–Romand medium (pH 5.5) in monoxenic culture. Germ tubes emerged through the spore wall in 88% of spores after 5 days. Hyphal contact with transformed linum roots was observed 5 days after co-cultivation. *Paris*-type arbuscules and hyphal coils were seen. Extra-radical branched absorbing structures were common. Terminal and intercalary secondary spores were also formed. Spore viability when assessed using vital dye staining (MTT test) was 83%. Secondary spores that proved viable were subsequently transferred from *in vitro* to *in vivo* culture where *Arum*-type arbuscules, intra-radical intercellular hyphae, and extra-radical spores were observed. The procedure established shows potential in AM inoculum mass production and possibility in application.

## Introduction

The arbuscular mycorrhizal (AM) association is a symbiotic union between the fungi of phylum Glomeromycota and plant roots. The phylum forms a monophyletic group of obligate mycobionts (Schüssler et al. 2001). In order to overcome the obligate biotrophic nature, AM fungal propagules are cultured in association with Ri T-DNA transformed hairy roots (Root Organ Culture technique). An advantage of using root organ culture as a system is that both symbionts (AM and host root) are grown and easily maintained on a defined medium, allowing nondestructive *in vivo* observations over long periods of time (de Souza and Berbara 1999). The system has afforded studies on organization and development of mycelium (Bago et al. 1998a), sporulation dynamics (Declerck et al. 2001, 2004; Voets et al. 2009; Ijdo et al. 2011), and spore ontogeny (de Souza et al. 2005). Further potential uses of the system are the production of pure and concentrated inoculum, and contaminant-free fungal tissues for genetic and physiological studies. Utilizing excised roots of a number of host species and various media formulations, the system has been developed to culture glomalean fungi monoxenically (Mosse and Hepper 1975; Miller-Wideman and Watrud 1984; Mugnier and Mosse 1987a, 1987b; Bécard and Fortin 1988). A large number of AM fungal species and isolates have been successfully maintained and sporulated in association with Ri T-DNA transformed roots of carrot (*Daucus carota* L.) (Fortin et al. 2002).

*Funneliformis mosseae* has been reported colonizing excised roots of tomato (*Solanum lycopersicum* L.) and clover (*Trifolium pratense* L.) (Mosse and Hepper 1975) and Ri T-DNA transformed roots of bindweed (*Calystegia sepium* (L.) R.Br.) and carrot (Mugnier and Mosse 1987b; Douds 1997). Mugnier and Mosse (1987a) reported successful establishment of *F. mosseae* on Ri T-DNA transformed roots of *C. sepium* in a two-compartment system. Roots were grown in nutrient medium in one compartment, crossing into a water agar and peat medium in the second compartment where they were inoculated with pre-germinated fungal spores. These authors further reported mycorrhizal dependence on the nitrogen (N) concentration of the nutrient medium in the root compartment for mycelium development. Douds (1997) manipulated buffer, pH and P levels to increase successful formation of mycorrhizae between carrot and *F. mosseae in vitro.* He reported that the cultures grew for 17–24 weeks before termination of the experiments, and new spores were not produced though the hyphae had spread throughout the Petri plates. Pawlowska et al. (1999) also reported absence of sporulation in *F. mosseae* while in dual culture. In our study, we report the successful establishment of endomycorrhizal association of *F. mosseae* with transformed roots of *Linum usitatissimum* L. (linum) on MSR media in monoxenic culture, and inoculum potential of *in vitro* produced spores to induce AM colonization *in vivo*.

## Materials and methods

### AM fungal isolate

The *F. mosseae* isolate used was obtained from Goa University Arbuscular Mycorrhizal Culture Collection (GUAMCC) wherein the fungus is propagated in pot cultures with *Solenostemon scutellariodes* L. (coleus) as host, grown in soil-sand (1:1) in a controlled environment greenhouse (25°C, RH 80–90%) with no supplementary lighting.

### Inoculum preparation

Spores of *F. mosseae* were extracted from soil by the Gerdemann and Nicolson (1963) wet sieving and decanting technique. Isolated spores were sterilized for 10 min in sodium hypochlorite, followed by a 10-min sterilization bath in an antibiotic solution (streptomycin sulfate 0.02% w/v and gentamycin sulfate 0.01% w/v) (Bécard and Fortin 1988). The two steps were followed by triple rinsing with sterile deionized water and the disinfected spores used as inoculum in the monoxenic culture.

### Culture medium

The modified Strullu–Romand (MSR) medium (Declerck et al. 1998) was used for both routine maintenance of the transformed roots and establishment of dual cultures.

### Root culture and *in vitro* establishment of monoxenic culture

The surface-sterilized spores were plated into MSR medium for germination, and the Petri plates were incubated in an inverted position in the dark at 27^o^C. Ri T-DNA transformed linum roots were also cultured on MSR medium and the Petri plates were similarly incubated. For the establishment of mycorrhizal association, an actively growing transformed linum root with several lateral branches was placed in the vicinity of the germinated spore and incubated in an inverted position in the dark at 27^o^C.

### Development of mycorrhizal symbiosis

Extra-radical hyphal growth and sporulation were monitored non-destructively using an Olympus BX 41 (100–1000x) microscope. Terminologies used to describe mycelium development are based on the Bago et al’s. (1998a) description of AM fungi *in vitro* development. To assess the AM colonization, the Phillips and Hayman (1970) trypan blue staining method was employed. Spore viability was estimated by the MTT 3-(4,5-dimethylthiazol-yl-2,5-diphenyl-2H-tetrazolium bromide) vital stain procedure (An and Hendrix 1988). Using separate cavity slides, 22 spores were treated with MTT for 40 h to allow maximum staining response. Treated spores were observed under a dissecting microscope. Spores staining red were considered viable and percent spore viability was recorded.

### Capacity of the *in vitro* produced spores to colonize plant roots *in vivo*

Coleus seeds were surface sterilized for 15 min using sodium hypochlorite (1–2%) solution, rinsed twice with sterile distilled water and plated on MSR media under sterile conditions. The seeds germinated in 7–14 days after plating. Germinated seeds were transferred to conical flasks containing sterilized vermiculite. The seedlings were maintained in a phytotron (Daihan Labtech, LGC-6201G) at 260 lux (16 h photoperiod), 26^o^C, 41.1% humidity and 100 ppm CO_2_, and fertilized with Hoagland’s solution (Hoagland and Arnon 1950) minus phosphorus every 20 days. Once the seedlings had established, they were inoculated with 20 *in vitro* produced *F. mosseae* spores and colonized (transformed) linum roots.

To isolate monoxenically produced spores, a fragment of gel containing sufficient spores with extra-radical mycelia was aseptically removed. To dissolve the gel under sterile conditions, 25 mL citrate buffer (0.01 M) was added (Cranenbrouck et al. 2005). The spores, along with attached extra-radical mycelia, were used as inoculum to colonize the *S. scutellariodes* seedlings. After 3 months, the roots were assessed for AM colonization (Phillips and Hayman 1970).

## Results

### Spore germination and hyphal growth

**Figure 1.**
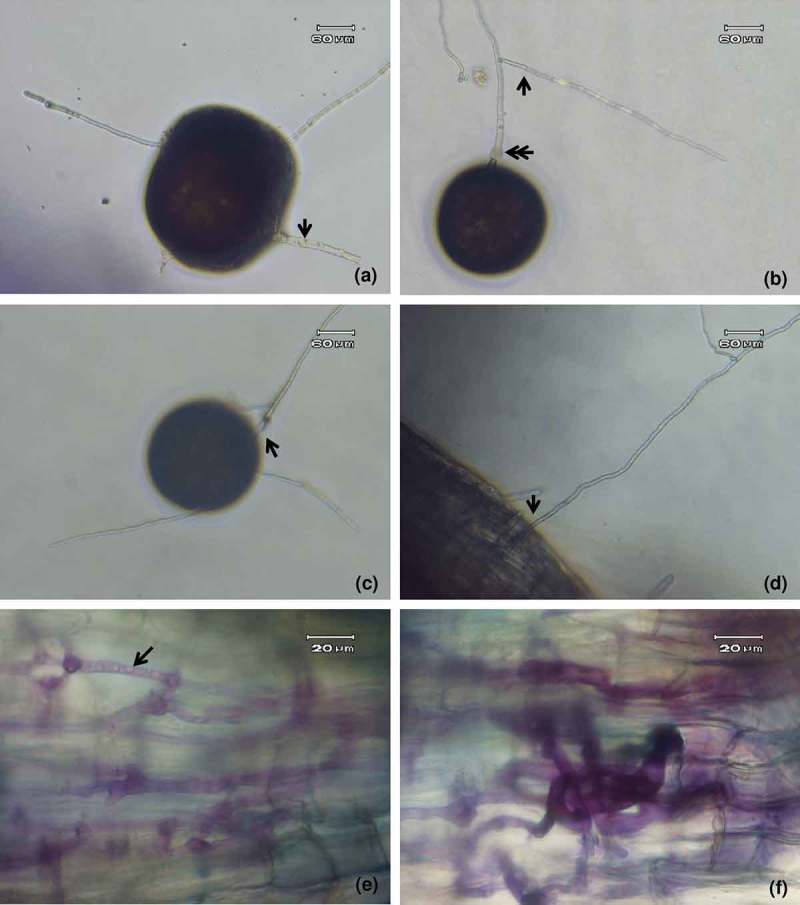
(a–f) *Funneliformis mosseae*: spore germination, hyphal contact and intra-radical root colonization in transformed linum roots.

Germ tube emergence was directly through the spore wall in majority (88%) of the spores (Figure 1a); in some (10%) of the spores, the germ tube emerged by re-growth through the broken end of the subtending hyphal attachment (Figure 1b). Two percent of the spores showed both modes of germination (Figure 1c). Spore germination initiated after 5 days of incubation and continued for 3 weeks. Multiple germ tubes were observed in some of the spores (Figure 1c). The germ tubes elongated, became septate and branched, as growth progressed (Figure 1b). The recorded germination rate was 86%. Germ tube length was measured every second day for a period of 6 days before association with the transformed roots and ranged between 194.4 and 388.8 µm. Average hyphal length was 291.6 ± 70.42 µm (*n* = 6) and diameter of 18 µm. The germ tubes were observed to be swollen at the base (Figure 1b). Several contact points between hypha and transformed roots were observed within 5 days of co-cultivation (Figure 1d).

#### Intra-radical root colonization of Ri T-DNA transformed linum roots by *F. mosseae*

The roots were excised, placed in a Petri dish and stained. Entry points were readily observed. Trypan blue staining indicated colonization levels of up to 85%. Almost all colonized cortex cells were penetrated by thick hyphae that spread mostly intracellularly (Figure 1e) and lipid bodies were clearly seen within. Hyphal coils (Figure 1f) and *Paris*-type arbuscules were observed, whereas vesicles were rarely observed.

#### *F. mosseae in vitro* sporulation and development of extra-radical mycelium

**Figure 2.**
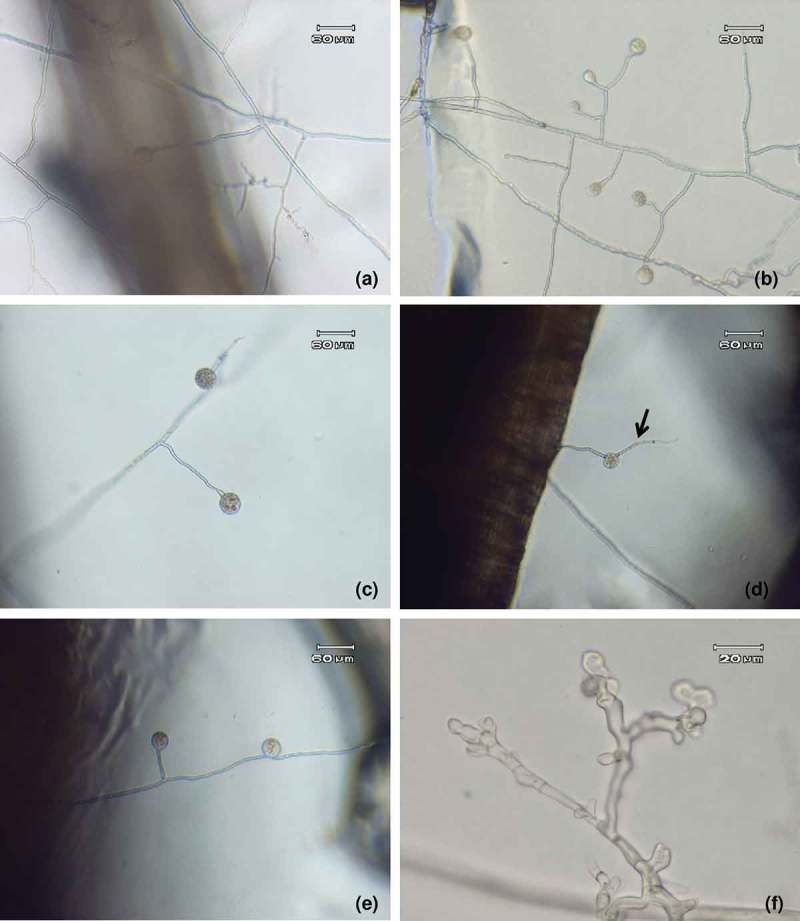
(a–f) *Funneliformis mosseae*: development of extra-radical mycelium and *in vitro* spore formation.

After 4–6 weeks of culture, mycelial biomass developed, and the agar surface was covered with an extra-radical mycelial network (Figure 2a). Numerous branched absorbing structures (BAS) were formed. The secondary spores were first observed in 14–21 days (Figure 2b). The spores formed, swelled and developed beyond the juvenile stage. Both mature and under-developed secondary spores were observed. As spore development progressed, color changed from hyaline to reddish-brown and contained numerous lipid droplets. Mean diameter was 102.5 µm (*n* = 6) where the mother spores had been 105–305 µm diameter. Intra-radical sporulation was also observed.

**Figure 3.**
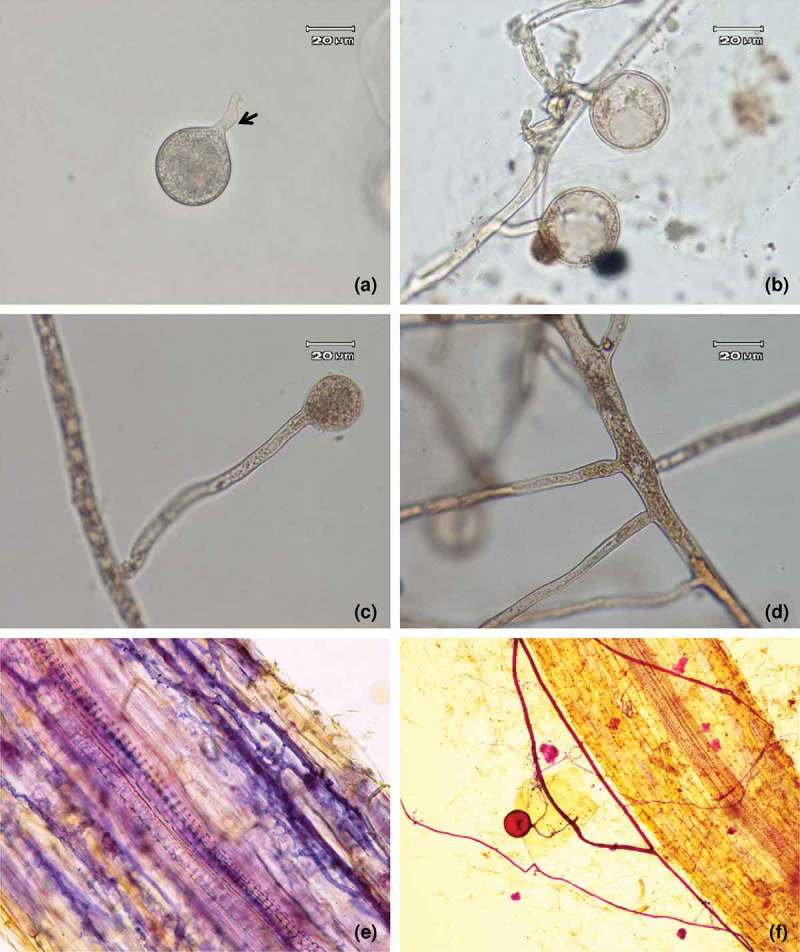
(a–f) *Funneliformis mosseae: in vitro* culture, viability of the culture by vital dye staining and *in vivo* root colonization by *in vitro* produced inocula in *Solenostemon scutellariodes.*

New secondary spores formed singly as terminal swellings on sporogenic hypha from first-order branching in the mycelial network that had originated from the primary spore (Figure 2c). Intercalary spore formation was also observed in the sporogenic hyphae (Figure 2d). In some dual culture plates, both patterns of sporulation were observed on a single sporogenic hypha (Figure 2e). Subtending hyphae were found as short lateral branches of the main hyphae. In several culture replicates, the primary hyphae bore arbuscule-like structures (ALS) or BAS. These were formed by a proliferation of thick, short, septate, hyphae that often developed into spore-like structures. The ALS often appeared in clusters (Figure 2f). In secondary spores, thickening of hyaline spore wall resulted in the formation of the layered wall. Spore contents coalesced into discernible oil droplets giving the spores an almost translucent appearance (Figure 3a). The generic morphological character of a funnel-shaped subtending hyphal attachment was clearly observed (Figure 3a). Growing hyphae exhibited bi-directional protoplasmic streaming which was observed as active movement of optically dense particles, under the microscope, during spore formation. The number of spores varied markedly between Petri dishes as: 24, 36, 225, and 300. Some of the spores did not remain viable throughout the experimental period and even showed loss of internal contents or coalescence of lipid droplets (Figure 3b).

#### Viability and re-infection capability of *in vitro* produced spores and colonized linum root fragments

Eighty-three percent of the spores from 90-day-old and 197-day-old cultures proved viable when assessed by vital dye staining (Figure 3c and d). Sub-culturing of dual cultures with media plugs containing *in vitro* formed spores and colonized root fragments showed active growth of hyphae from the media plugs in seven of the dual culture plates.

#### Capacity of *in vitro* produced spores to colonize plant roots *in vivo*

Trypan blue staining indicated percentage root colonization levels of 63.66% ± 4.04 (*n* = 3) after 90 days. The presence of intercellular hyphae (Figure 3e) and *Arum*-type arbuscules in stained roots, and extra-radical spores (Figure 3f), indicated successful colonization of *S. scutellariodes* by the *in vitro* produced uncontaminated inocula of *F. mosseae.*

## Discussion

The germ tube of *F. mosseae* normally emerges by re-growth through the broken end of the hyphal attachment (Nicolson and Gerdemann 1968). In this study, the germ tubes emerged directly through the spore wall in 88% of the spores and 10% emerged by re-growth through broken end of the subtending hyphal attachment, whereas in 2% both modes of germination were observed. Direct germination has been reported in only a few Glomeraceae species *viz., Glomus albidum* (Walker and Rhodes 1981), *Glomus caledonicum*, and *G. monosporum* (Tommerup and Kidby 1980) and considered uncommon in all Glomeraceae species except *G. albidum* (Meier & Charvat 1992). *Paraglomus* species (*Pa. occultum*, *Pa. brasilianum*, and *Pa. lacteum*) also form glomoid spores that germinate directly through the spore wall (Oehl et al. 2011). The germ tube was observed to be swollen at the base, similar in appearance to germ tubes of *Gi. margarita* (Sward 1981), and this could be interpreted as the result of increased pressure on the germ tube wall by the emerging cytoplasm (Meier & Charvat 1992). Spore germination directly through the spore wall was recorded in the majority of the spores after 5 days in MSR medium (pH 5.5). Budi et al. (1999) observed a high percentage of germination after 21 days in 0.6% water agar. Douds (1997) observed germination up to 80% after 25 days in 10 mM Tris (pH 7.3–7.6) and 10 mM MES (pH 6.7) and 60–70% germination in a shorter time period, (14–20 days) in 10 mM Tris, pH 7.1–7.3. The multiple germ tubes observed in a number of spores may be a survival strategy whereby the chance of contact with a compatible host gets increased (Maia et al. 2010; Costa et al. 2013).

Formation of *Paris*-type arbuscules was observed in the colonized transformed linum root fragments. It was reported that arbuscules of *F. mosseae* (Mosse and Hepper 1975) and *Gi. margarita* (Miller-Wideman and Watrud 1984) produced in older roots in monoxenic cultures remained vestigial and looked ‘stumpy’, with few fine branches. The main feature was massive, considerably swollen hyphae growing longitudinally within the intercellular spaces (Mosse and Hepper 1975). In this study, intra-radical hyphal spread was mostly intracellular, with visible coils of thick hyphae within the cells. Bi-directional cytoplasmic flow in the thick sporogenous and subtending hyphae was also observed. Karandashov et al. (2000) reported that *G. caledonium* formed a *Paris*-type mycorrhiza on transformed carrot roots and suggested that carrot has an unclear status with respect to the formation of *Arum*- or *Paris*-type mycorrhiza, whereas in our study, *F. mosseae* formed a *Paris*-type mycorrhiza on transformed linum roots. In natural environment, *F. mosseae* is known to form *Arum*-type mycorrhiza in many plant species, but *Paris*-type mycorrhiza in others, as exemplified in *Smilax aspera* L. (Bedini et al. 2000). This suggests that the formation of *Arum*- or *Paris*-type mycorrhiza may be primarily under the genetic control of the host plant (Smith and Smith 1997).

Douds (1997) reported *F. mosseae* growth on minimal (M) medium, which colonized transformed carrot roots but failed to sporulate. Germination was observed only when the pH of M medium was buffered above pH 7.0. In other studies, development of *F. mosseae* (Mosse and Hepper 1975; Mugnier and Mosse 1987a) and *Rhizophagus irregularis* (Mosse 1988) was inhibited at low pH and hyphae grew only after the pH was raised. MSR medium has a relatively low pH (5.5) and is routinely used in GINCO (Glomeromycota *in vitro* Collection) for monoxenic cultivation of AM fungi (Cranenbrouck et al. 2005). In the present study, the germination rate of *F. mosseae* spores and formation of colonization in the presence of growing linum roots was not mal-affected by MSR media composition or pH (5.5). This suggests that the early mycorrhizal developmental events, such as spore germination, germ tube growth, host recognition and initial root contact, determine the success or failure of the AM symbiosis.

The MSR medium proved highly suitable for dual culture of *F. mosseae* and transformed linum roots. The levels of root colonization and sporulation achieved were considerably high, and mycorrhizal development was established after successful root colonization. The number of penetration points initiated by the germinating hyphae resulted in extensive mycelial development, leading to rapid sporulation in minimum cultivation time. Intra-radical spore formation was also observed, in our study. The secondary spores produced averaged 102.5 µm diameter, similar to soil-borne spores described by Gerdemann and Trappe (1974) that had an average size of 105–310 × 110–305 µm. The characteristic funnel-shaped hyphal attachment was also observed at the base of the *in vitro* formed spores which is the defining feature of soil-borne spores (Nicolson and Gerdemann 1968). As mentioned earlier, secondary spore contents coalesced into discernible hyaline oil droplets, giving the spores a translucent appearance. Young spores contain small lipid droplets that are known to coalesce with maturity (Mosse 1959).

Spores were formed singly as terminal swellings on sporogenic hypha and at the intercalary position in the sporogenic hypha. In some of the culture plates, both patterns of sporulation were observed on a single sporogenic hypha. Both patterns of spore formation have been reported for *G. versiforme* and *R. irregularis* in root organ culture (Chabot et al. 1992; Bonfante and Bianciotto 1995; Declerck et al. 1996; Bago et al. 1998a, 1998b). The development of extra-radical hyphae was accompanied by the formation of BAS or ALS (Mosse and Hepper 1975; Bécard and Fortin 1988; Chabot et al. 1992; Bago et al. 1998a, 1998b). It was noted that BAS were composed of thick, branched, septate hyphae. It has been suggested that the occurrence of these structures is a specific response to close interaction with the root (Bécard and Fortin 1988), or a localized response to an unknown stimulus within the medium (Mosse and Hepper 1975). Bago et al. (1998a, 1998b) suggested that ALS/BAS may play an important role as preferential sites for nutrient acquisition by the extra-radical mycelium.

In this study, it was found that sporulation did not follow the standard three-phase development (lag, exponential, and plateau) common to most biological populations, including AM fungi, which was observed in similar earlier studies (Declerck et al. 1996; Bago et al. 1998a). A variation in the time duration of spore production was observed. This may be a feature of the fungal isolate or may be due to the effect of culture conditions on the isolate. To investigate this phenomenon, pot experiment determining sporulation rates at different times needs to be performed.

The spores produced in the *in vitro* culture retained moderate inoculum potential when sub-cultured and were successfully inoculated in *in vivo* plant roots to form mycorrhizal association. Up to 90 days, the majority of the spore primordia tested positive for viability and the level remaining unchanged throughout the spore maturation period (90–197 days). As the spores were produced *in vitro* under uniform culture conditions and with no growth constraints, the fungal material may have been less susceptible to mortality when compared to spores isolated directly from soil. Sub-culture of the media plugs containing *in vitro* formed spores and colonized root fragments showed active growth of hyphae from the media plugs in seven of the ten dual culture plates.

*In vitro* produced inocula (spores and colonized roots) of *F. mosseae* exhibited hyphal and *Arum*-type root colonization and produced extra-radical spores in *S. scutellariodes.* Vimard et al. (1999) reported AM colonization by monoxenically produced *R. intraradices* spores in *Allium porrum* L. (leek) grown in sterilized soil mix and calcined montmorillonite clay.

## Conclusions

The main understandings emerged from this work on endomycorrhizal association of *F. mosseae* with transformed roots of linum include spore germination through spore wall and broken end of subtending hypha, root colonization, intra-radical sporulation, development of extra-radical mycelium and branched absorbing structures, and extra-radical sporulation through terminal and intercalary patterns on MSR medium pH 5.5 with no supplementary source. The capability of viable monoxenically produced spores to induce AM colonization *in vivo* was demonstrated successfully, indicating suitability of the method for large-scale inoculum production.

## Disclosure statement

No potential conflict of interest was reported by the authors.
